# Auditory distance perception in humans: a review of cues, development, neuronal bases, and effects of sensory loss

**DOI:** 10.3758/s13414-015-1015-1

**Published:** 2015-11-20

**Authors:** Andrew J. Kolarik, Brian C. J. Moore, Pavel Zahorik, Silvia Cirstea, Shahina Pardhan

**Affiliations:** Centre for the Study of the Senses, Institute of Philosophy, University of London, Senate House, Malet Street, London, WC1E 7HU UK; Vision and Eye Research Unit (VERU), Postgraduate Medical Institute, Anglia Ruskin University, Eastings 204, East Road, Cambridge, CB1 1PT UK; Department of Psychology, University of Cambridge, Downing Street, Cambridge, CB2 3EB UK; Department of Psychological and Brain Sciences, University of Louisville, Louisville, KY 49292 USA

**Keywords:** Distance perception, Blindness, Hearing loss, Sound level, Compensatory plasticity

## Abstract

Auditory distance perception plays a major role in spatial awareness, enabling location of objects and avoidance of obstacles in the environment. However, it remains under-researched relative to studies of the directional aspect of sound localization. This review focuses on the following four aspects of auditory distance perception: cue processing, development, consequences of visual and auditory loss, and neurological bases. The several auditory distance cues vary in their effective ranges in peripersonal and extrapersonal space. The primary cues are sound level, reverberation, and frequency. Nonperceptual factors, including the importance of the auditory event to the listener, also can affect perceived distance. Basic internal representations of auditory distance emerge at approximately 6 months of age in humans. Although visual information plays an important role in calibrating auditory space, sensorimotor contingencies can be used for calibration when vision is unavailable. Blind individuals often manifest supranormal abilities to judge relative distance but show a deficit in absolute distance judgments. Following hearing loss, the use of auditory level as a distance cue remains robust, while the reverberation cue becomes less effective. Previous studies have not found evidence that hearing-aid processing affects perceived auditory distance. Studies investigating the brain areas involved in processing different acoustic distance cues are described. Finally, suggestions are given for further research on auditory distance perception, including broader investigation of how background noise and multiple sound sources affect perceived auditory distance for those with sensory loss.

The ability to judge the distance of sounds is important for building up a representation of the environment and for the interpretation of those sounds. Audition is the main means of evaluating distance when vision is degraded, due to environmental or physiological factors, or when the sound-producing object is outside of the visual field. In contrast to light, sound is generally able to travel around occluding objects. Thus, audition provides us with important cues when evaluating the distance of objects that are not visible. Whereas touch can only provide spatial information for objects within reaching and grasping distance, the auditory modality can be used to detect and judge objects that are farther away from the listener. Furthermore, audition plays a key role in guiding locomotion by the central nervous system (CNS) when vision is not available, for which an accurate internal representation of the distance between the organism and the target is essential. However, auditory estimates of distance are generally poorer than those for azimuth (left-front-right judgments; Middlebrooks & Green, 1991).

A distinction can be made between sounds in *peripersonal space*, i.e., sounds that are within reaching and grasping distance (approximately 1 m from the listener) and farther sounds in *extrapersonal space*. This distinction is useful because the range over which distance cues are operable varies, and some cues are only useful within peripersonal space, a region where internal representations of distance are based on both auditory and tactile information (Serino, Canzoneri, & Avenanti, 2011). Peripersonal space is a region especially relevant to behavior. Many important everyday events, such as personal conversations, occur with sound sources that are close to the listener, and an appropriate selection of a target voice from a mixture of voices may require accurate spatial information (Shinn-Cunningham, Kopčo, & Martin, 2005). Nearby auditory events may require immediate motor responses, especially if the signal is threatening or particularly interesting (Serino, et al., 2011), and accurate auditory distance information is needed to coordinate this.

The issue of how auditory space is generated, calibrated, and maintained when vision or hearing are impaired is of considerable interest in neuroscience and psychology (Collignon, Voss, Lassonde, & Lepore, 2009; Gori, Sandini, Martinoli, & Burr, 2014; Lewald, 2002b, 2013; Voss et al., 2004). A current question is how the external world is internally represented in blind people, who cannot use visual information to calibrate auditory space. A large body of evidence shows that severe visual loss leads to an enhancement of directional localization abilities, especially for signals located in peripheral space (Doucet et al., 2005; Gougoux, Zatorre, Lassonde, Voss, & Lepore, 2005; Lessard, Pare, Lepore, & Lassonde, 1998; Simon, Divenyi, & Lotze, 2002). These enhanced abilities often are coupled with cortical reorganization, such that visually deafferented brain regions within the occipital cortex are recruited to process auditory input (for reviews, see Voss, Collignon, Lassonde, & Lepore, 2010; Voss & Zatorre, 2012). The effect of visual loss on auditory distance perception is considerably less clear, due in part to the sparse number of behavioral studies on this topic and the scarcity of neural data. It is still largely unknown whether visual loss leads to cortical reorganization that affects auditory distance perception, although recent work involving distance-to-sound learning with sensory substitution devices (SSDs) suggests that occipital areas are recruited for auditory distance processing following visual loss (Chan et al., 2012; Tao et al., 2013). The literature on the effects of sensory loss on auditory distance perception has not previously been reviewed and is discussed below. We discuss evidence suggesting that visual loss systematically affects auditory distance perception, thereby leading to decreased abilities to judge absolute auditory distance but enhanced abilities to judge relative distance. We argue that severe visual loss distorts internal spatial representations of the environment while enhancing abilities to discriminate between sound sources.

In this review, we examined the psychophysical and neuronal bases of human auditory distance perception, and the effects of sensory loss. We first describe the various acoustic cues that are used to perceive distance and the non-acoustic factors that influence this. A summary of research investigating the development of auditory distance perception is presented. The means by which auditory distance is calibrated in peripersonal and extrapersonal space and its effectiveness for guiding locomotion are reviewed. Findings of studies that have investigated the effects of visual and auditory loss on auditory distance perception are summarized. Research that has explored the neural processes associated with auditory distance is described. Finally, we highlight potential avenues for future research relevant to auditory distance perception and the impact of sensory loss.

## Perceiving distance using sound

Knowledge about the processing of auditory distance cues has been advanced by the development of binaural technology that allows simulation of different acoustical environments via headphone presentation (Zahorik, 2002a). Such technology allows realistic simulation of sounds presented from different distances for various listener positions. It also allows auditory distance cues to be manipulated independently in a controlled way. This technology was used in many of the studies described below.

**Fig. 1:**
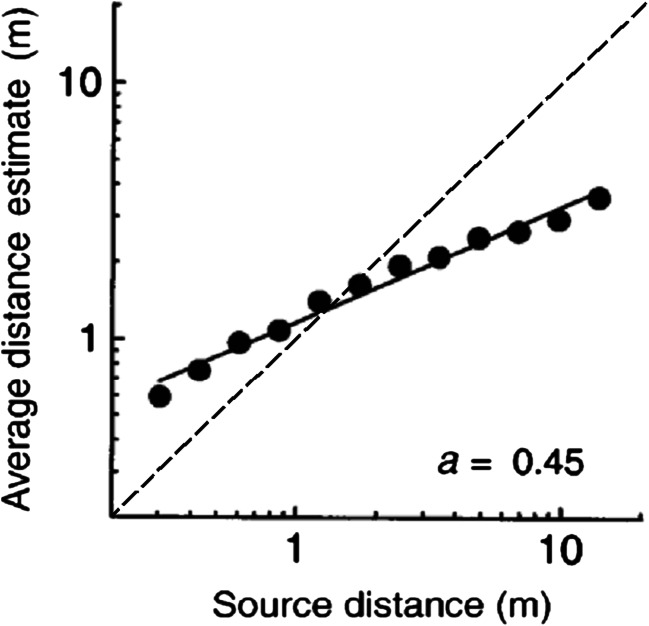
Average apparent distance estimates (10 estimates/distance/participant, n = 5) plotted as a function of sound source distance. A power function was fitted to the data, and the exponent, *a*, which on double-logarithmic coordinates equals the slope of the linear fit, is reported in the bottom right. The dashed diagonal line indicates where veridical judgments would lie. Adapted from “Loudness constancy with varying sound source distance,” by Zahorik and Wightman 2001, *Nature Neuroscience*, *4*, p. 81. Copyright 2001 by Nature Publishing Group. Reprinted with permission

On average, perceived distance to sound sources in peripersonal space tends to be overestimated, while distance to sounds in extrapersonal space is generally underestimated for normally sighted and hearing humans (Fontana & Rocchesso, 2008; Kearney, Gorzel, Rice, & Boland, 2012; Parseihian, Jouffrais, & Katz, 2014; Zahorik, 2002a; Zahorik, Brungart, & Bronkhorst, 2005; Zahorik & Wightman, 2001). This is illustrated in Fig. 1, which shows distance judgments for noise bursts presented at virtual distances (via a headphone simulation) between 0.3 and 14 m. More veridical judgments are made when close sound sources are presented laterally relative to the listener (Kopčo & Shinn-Cunningham, 2011). This is contrary to azimuthal localization, which is generally more accurate for sources near the midline (Middlebrooks & Green, 1991). Auditory distance judgments are generally most accurate for sound sources approximately 1 m from the listener. Zahorik et al. (2005) demonstrated that systematic biases in distance estimates occur across a wide range of stimulus conditions, acoustic environments, and psychophysical procedures. Based on previous findings, they showed that compressive power functions of the form *r*′ = *kr*^*a*^ gave good fits to distance judgments, where *r*′ is the judged distance, *r* is the actual distance, and *k* and *a* are adjustable parameters (with *a* < 1). Such systematic biases are perhaps surprising, given that humans have a tacit knowledge of physical sound propagation losses with increasing distance. This is indicated by the ability to compensate for these losses with fairly accurate adjustment of vocal output level, suggesting that auditory distance processes are to an extent separate from vocal compensation processes (Zahorik & Kelly, 2007). It also is noteworthy that such biases do not occur for visual depth perception, where under natural full-cue conditions distance estimates are highly accurate (Da Silva, 1985).

In addition to being biased, auditory distance estimates appear to be considerably less precise than visual distance estimates. This reduction in precision, or distance “blur,” is evident in the considerable variability often observed in (un-averaged) auditory distance estimates. For example, Anderson and Zahorik (2014) reported that the average standard deviation of sound source distance estimates was approximately 1.6 times the distance of the target. This corresponds to nearly twice the variability observed for comparable estimates of distance to visual targets (Anderson & Zahorik, 2014).

There are multiple acoustic cues available for perceiving the distance between a listener and a sound source. The number of cues available and their reliability can vary substantially depending upon the stimulus, the properties of the environment, and the direction of the sound source. Two types of auditory distance cues can be distinguished. Absolute cues allow distance to be judged based on single presentations of sounds to independent groups of listeners (Mershon, Ballenger, Little, McMurtry, & Buchanan, 1989). Relative cues allow sounds at different distances to be discriminated. In addition, there is now a considerable body of work showing that visual information and nonperceptual factors can influence estimates of perceived distance. Zahorik, et al. (2005) and Coleman (1963) previously reviewed the auditory distance cues used by humans, and Naguib and Wiley (2001) summarized the use of auditory distance cues by humans and animals. In the following sections, we summarize work that has investigated the cues used for auditory distance perception by normally sighted and hearing humans.

### Level

Overall level is a relative distance cue that is available in most environments (Ashmead, LeRoy, & Odom, 1990; Coleman, 1963; Gamble, 1909; Gardner, 1969; Mershon & King, 1975; Strybel & Perrott, 1984; Zahorik, 2002a) and is effective over a wide range of distances. Perceived source distance generally increases with decreasing level of the sound at the ears of the listener (receiver). In an anechoic environment, the relationship between level and distance between a sound source and receiver is characterized by the inverse-square law, and level falls by approximately 6 dB for each doubling of the source distance (Coleman, 1963). The rate of decrease of level is somewhat reduced in reverberant environments. For example, in an auditorium used by Zahorik (2002a), the rate was approximately 4 dB/doubling. The rate of change also depends on the directivity of the sound source, i.e., on whether the source radiates uniformly in all directions or produces sound more like a “beam.”

Level is a relative distance cue, as distance judgments made solely on the basis of level may be confounded by variation in the level at the source (Zahorik et al., 2005). This can make absolute distance judgments for single sound sources based solely on level difficult or impossible, although relative distances of two or more sound sources can be judged. When level is the primary cue available, the perceived distance to a single sound source generally increases at a lower rate than the physical distance when the source distance is greater than 1 m (Cochran, Throop, & Simpson, 1968; Simpson & Stanton, 1973; von Békésy, 1949). For relative distance perception, the *pressure*-*discrimination hypothesis* posits that just-noticeable-differences in source distance are determined by the ability to discriminate changes in sound pressure. Miller (1947) found that, for broadband noise, the smallest detectable change in level was approximately 0.4 dB, whereas for sinewaves the threshold is typically 1-2 dB, depending on frequency and sound level (Jesteadt, Wier, & Green, 1977; Riesz, 1933). This leads to predicted thresholds for distance discrimination ranging from approximately 5% to 25% of the reference distance, depending on the type of stimulus. Consistent with this, Ashmead et al. (1990) found that the threshold change in distance for a white noise burst was approximately 6%. Most other studies in which level was the primary cue, using a variety of stimuli, have revealed higher thresholds. Threshold changes in distance were reported to be approximately 20% by Edwards (1955) and Gamble (1909), 13% by Simpson and Stanton (1973), and 25% by Akeroyd, Gatehouse, and Blaschke (2007). The differences across studies may be due to a number of factors, including stimulus differences, the environment, the presence of distance cues apart from level, and differences in the reference distance.

While level provides information regarding sound-source distance, the auditory system cannot simply relate the level at the listener’s ears to distance, because the received level is dependent on the acoustic power of the sound source and its directivity. When judging loudness, listeners appear to estimate the power of the source, because a source with a fixed power located at different distances is judged to have a constant loudness (Altmann et al., 2013; Zahorik & Wightman, 2001). This has been called “loudness constancy.” However, such constancy typically only occurs when cues other than level are available. It has been suggested that sound-source power is estimated from the reverberant sound energy, which remains approximately constant across distances in indoor environments (Zahorik & Wightman, 2001). This hypothesis was supported by Altmann et al. (2013), who found that loudness constancy was generally found in a room with strong reverberation (*T*60 = 1.03 s, where *T*60 is the time taken for the signal level to decay by 60 dB) but not in a room with weak reverberation (*T*60 = 0.14 s), whereas distance judgments were similar across different room reverberation times.

### Direct-to-reverberant energy ratio (DRR)

For localization in azimuth, reverberation degrades performance (Hartmann, 1983). However, the presence of reverberation for distance judgments is beneficial, as DRR is an important cue for judging sound-source distance (Bronkhorst & Houtgast, 1999; Kopčo & Shinn-Cunningham, 2011; Mershon, et al., 1989; Mershon & King, 1975; von Békésy, 1938; Zahorik, 2002a, 2002b). The DRR decreases as source distance from the listener increases, and this is associated with increasing perceived distance. Direct sound energy travels in a straight line from the source to the listener, and, for an omni-directional source, its level falls by 6 dB for each doubling of the source distance. Reverberant sound energy is reflected from surfaces, such as walls or objects, before reaching the listener and can be approximated by a diffuse sound field with constant energy regardless of source location if the room is not too small; the level of the reverberant sound varies only slightly with distance (Zahorik, 2002a). For example, in the small auditorium utilized by Zahorik (2002a), the level of the reverberant sound reduced by only about 1 dB for each doubling of the source distance. The magnitude of the reverberant energy is determined by the room size and shape and by the absorption coefficients of the walls, floor, and ceiling and the objects in the room. DRR has been demonstrated to provide absolute distance information (Mershon & King, 1975) and is primarily useful in indoor environments. However, outdoor environments also can produce reverberation (Richards & Wiley, 1980), although it is unknown whether this provides an effective distance cue for humans. For sounds near to the listener, Kopčo and Shinn-Cunningham (2011) showed that participants judged distance using a fixed DRR-to-distance mapping that varied with frequency but was direction-independent, using the DRR at the ear nearest to the sound source.

Like level, DRR is an effective distance cue for sound sources in peripersonal and extrapersonal space, for both frontal and lateral sounds. Level cues in isolation generally provide more accurate information than DRR only in isolation (Zahorik, et al., 2005), although level and DRR cues can provide equally accurate information for discriminating distance in highly reverberant environments (Kolarik, Cirstea, & Pardhan, 2013a). Distance perception is generally most accurate when both DRR and level cues are available (Bronkhorst & Houtgast, 1999; Kopčo & Shinn-Cunningham, 2011; Nielsen, 1993; Ronsse & Wang, 2012).

The amount of reverberation may affect judgments of distance. Mershon et al. (1989) reported that distance estimates made in a room with a long reverberation time were greater than those obtained in a room with a short reverberation time. Altmann et al. (2013) obtained distance judgments using both headphone stimulation and loudspeaker presentation, in separate experiments, and found for both methods that increasing the reverberation time marginally increased the judged distance of remote sources, although not significantly. They attributed this finding to the relatively short reverberation time (called *T*60) used in the “strong reverberation” condition of their study (*T*60 at 500 Hz = 1.03 s), whereas for the “live room” of Mershon et al. (1989) *T*60 was 2.17 s. It also is possible that the instructions informing participants of the maximum possible distance may have limited the overall range of their responses in the study of Altmann et al. (2013). Guski (1990) varied the location of a single reflective surface within an anechoic room and reported that the reflections from the surface did not affect auditory distance estimates. This is perhaps not surprising, because the reflective surface added only a single echo and minimal reverberant energy, so the DRR remained an ineffective distance cue regardless of the presence of the surface.

For ongoing sounds, it is unlikely that listeners are able to segregate the direct and reverberant sound to compute DRR directly, and instead probably utilize co-varying physical characteristics of the signal (Kopčo & Shinn-Cunningham, 2011), such as changes in spectrum or time pattern (Larsen, Iyer, Lansing, & Feng, 2008). Larsen et al. (2008) reported that DRR sensitivity changed depending on the reference DRR value. Using bursts of white noise as the stimulus, sensitivity was highest around the critical distance, which is the distance at which the direct and reverberant sounds have equal energy and DRR = 0 dB (Jetzt, 1979). Sensitivity was lower for high and low DRR values, corresponding to situations where sounds were considerably closer to or farther from the listener. For wideband noise bursts, DRR just-noticeable-differences (JNDs) were 2–3 dB at 0 and +10 dB DRR and approximately 6–8 dB at −10 and 20 dB DRR. Reducing the stimulus bandwidth reduced spectral variance and spectral envelope cues without affecting temporal cues (buildup or decay times of sounds at the ear canal) and increased JNDs by 1.5 dB at a DRR of 0 dB, suggesting that spectral cues were needed for high DRR sensitivity. When spectral envelope cues were removed by roving the center frequency of a narrowband signal, listeners were still capable of discriminating DRR, although JNDs increased by 1.6 dB at a DRR of 0 dB, suggesting that temporal cues may be used when spectral cues are missing or degraded. Results for wideband noise were in partial agreement with a previous study of Reichardt and Schmidt (1966), who found that, for stimuli consisting of classical music, JNDs were 2 dB at 0 dB DRR and were approximately 20 dB at ±20 dB DRR. Conversely, a study of Zahorik (2002b) suggested that sensitivity to changes in DRR was approximately equal across a range of positive DRRs for stimuli composed of speech, noise, or an impulse presented frontally and laterally. He reported JNDs of 5-6 dB for DRR values between 0 and 20 dB. This discrepancy may be due to differences in experimental procedure and stimuli. However, the acoustical analysis performed by Larsen et al. (2008) showed that relevant acoustical variables, including spectral variance and spectral envelope and temporal buildup/decay times, reach asymptotic values for large positive and negative DRR values. This suggests that sensitivity to changes in DRR should decline for very low and high DRR values.

The analysis of reverberant sound fields by Larsen et al. (2008) showed that acoustic properties thought to be relevant to DRR processing, including interaural cross-correlation (a measure of the similarity of the signals received at the two ears), spectral, and temporal cues, become relatively constant at large source distances. Larsen et al. described this as an “unavoidable property of room acoustics…responsible for the auditory horizon effect.” This effect has been proposed to impose a limit upon the maximum perceived auditory distance (Bronkhorst & Houtgast, 1999; von Békésy, 1949), consistent with underestimation of the distance of sounds in far space. However, the limit imposed by the auditory horizon has not yet been measured directly, and underestimation of sound source distance occurs well before the maximum judged distance is reached (Fig. 1).

Kim, Zahorik, Carney, Bishop, and Kuwada (2015) investigated whether DRR information could be converted into a neural signal coding sound distance using monaural amplitude modulation (AM) depth as a cue. Reverberation results in a reduction of AM depth in sounds (Kuwada, Bishop, & Kim, 2014), and the reduction increases as distance increases, due to the decrease in DRR. Inferior colliculus (IC) neurons in the midbrain show sensitivity to AM depth; some neurons increase their firing rates as AM depth increases, whereas other reduce their rates (Joris, Schreiner, & Rees, 2004; Krishna & Semple, 2000; Nelson & Carney, 2004). Kim et al. (2015) and Zahorik and Anderson (2014) showed that normally hearing listeners could judge distance for 1-octave wide noise bands centered at 4 kHz presented up to 2 m away when monaural AM cues were available, and level cues were made unavailable, in reverberant but not anechoic environments. These findings parallel neural responses in the rabbit (Kim et al., 2015), suggesting that AM depth may function as a DRR-related auditory distance cue.

### Spectral cues

Spectral shape can be used to perceive the distance to sound sources more than 15 m from the listener (Blauert, 1997) and also to sounds in peripersonal space (Brungart, 1999; Kopčo & Shinn-Cunningham, 2011). For far away sources, as sound travels through air higher frequencies become more attenuated than lower frequencies, altering the spectral shape. Sounds with decreased high-frequency content relative to low-frequency content are perceived to be farther away (Butler, Levy, & Neff, 1980; Coleman, 1968; Little, Mershon, & Cox, 1992; von Békésy, 1938). Butler et al. (1980) recorded broadband, low-pass (cutoff frequencies of 2.0, 1.0, or 0.5 kHz) and high-pass (cutoff frequencies of 6.0, 4.0, and 2.0 kHz) noise in the ear canals of humans in an anechoic or reverberant room. The sounds were played back to participants over headphones. Low-pass noises were consistently judged to be farther from the participants than high-pass noises recorded in both anechoic and reverberant rooms, and the broadband noise was judged to originate in the middle of the overall range of perceived distances. Little et al. (1992) utilized shaped broadband noises low-pass filtered at 5, 6, and 6.7 kHz, which they argued were more ecologically appropriate stimuli, as the substantially different spectral contents of the sounds used by Butler et al. (1980) could not be produced by physical changes in distance to a sound source. Decreases in high-frequency energy were associated with greater judged distance, but only over the course of several trials, suggesting that sound spectrum is a relative distance cue.

Spectral content also is important in perceiving distance to nearby sounds, due to the way that diffraction of sound around the head varies with frequency and distance. Brungart (1999) obtained distance judgments in an anechoic chamber for broadband (0.2–15 kHz), high-passed (3–15 kHz), or low-passed (0.2-3 kHz) noise bursts. Accurate distance judgments for proximal sound sources required components below 3 kHz. In a study by Kopčo and Shinn-Cunningham (2011), participants judged the distance of noise bursts at distances between 0.15 and 1.7 m that varied in center frequency between 300 and 5700 Hz, and in bandwidth between 200 and 5400 Hz. The sounds were presented in a reverberant environment and the level was roved to make it an unreliable cue. The accuracy of distance judgments decreased for both frontal and lateral sounds as low-frequency energy was removed from the signal, although bandwidth did not affect the mean distance judgments. The effect of spectrum was strongest for the frontal sounds. Judgments were relatively accurate when sounds contained energy at frequencies around 300 Hz and were less accurate for sounds with energy only at 5700 Hz.

Note that spectral cues do not provide distance information for sounds located in the range 1-15 m from the listener, for which the sound has not traveled far enough to have lost a detectable amount of energy at higher frequencies and the low-frequency cues provided by diffraction around the head are too small to be detected.

Evidence from a study by Gordon, Russo, and MacDonald (2013) showed that source spectrum also can affect perceived distance when the sound source is moving. Approaching sounds were simulated by applying naturalistic changes in sound level for fast, medium and slow speeds to nine 1-octave-wide noise bands, with center frequencies ranging from 60 Hz to 15 kHz. Time to arrival was generally underestimated. Errors were smallest for bands with center frequencies between 120 and 250 Hz and underestimation was highest for sounds with center frequencies between 2000 and 7500 Hz. The authors suggested that higher-frequency content was associated with greater underestimation due to the increased perceived urgency of high-frequency sounds. The finding that the presence of low-frequency components led to smaller underestimation of time to arrival (hence sounds were perceived to be farther away) is consistent with previous studies using static sounds, showing that stimuli with relatively weak high-frequency content were judged to be farther from the participant (Butler et al., 1980; Little et al., 1992).

### Binaural cues

For close sound sources, auditory distance judgments tend to be more accurate when the sound is presented laterally relative to the listener (Kopčo & Shinn-Cunningham, 2011). This is due to the added benefit of binaural cues which are noticeable even in the presence of prominent level and DRR cues. When sounds are heard laterally or when the listener turns their head, the signal at the ear farther from the source is attenuated and delayed. This produces interaural level differences (ILD) and interaural time differences (ITD) between the ears. Although ITD changes are approximately independent of distance, ILD changes substantially as a function of distance in the acoustic near-field (Brungart, Durlach, & Rabinowitz, 1999; Duda & Martens, 1998). ILD provides a distance cue for distances up to approximately 1 m, beyond which it becomes roughly independent of source distance (Brungart, et al., 1999; Greene, 1968). In particular, ILDs for low-frequency sounds can be large for nearby sources but are very small for distant sources.

Theoretical work by Hartley and Fry (1921) suggested that the distance of a pure tone could be estimated at near distances using ILD information. However, an experiment of Wightman and Firestone (1930) showed that listeners were unable to judge the distances of pure-tone stimuli. Hirsch (1968) theorized that listeners could combine information from ILDs and ITDs to determine source distance. Molino (1973) modified Hirsch’s theory so that it would apply to cases where the source direction was known, but again found that listeners could not make distance judgments for pure-tone stimuli. Duda and Martens (1998) suggested that the use of pure tones may have resulted in the stimuli not being heard as external to the listener’s head, potentially explaining why distance judgments were difficult for pure-tone stimuli. Only small benefits of head movements that introduced binaural cues were reported by Gardner (1969) for judging distances to speech in an anechoic room. Holt and Thurlow (1969) reported that listeners were not able to judge the distance of thermal noise (similar to white noise) presented frontally at distances beyond 1.8 m, but performance improved when the sound sources were oriented laterally. Cochran et al. (1968) found that the orientation of the head had no effect on distance judgments for speech presented at distances greater than 1 m, and Simpson and Stanton (1973) found that head movements did not aid judgments of distance for pulse trains at distances between 0.3 and 2.66 m.

The binaural cues available at low frequencies, as measured using the head-related transfer function (HRTF, the transfer function from a sound source to the eardrum of the listener) have been approximated by modelling the human head as an ideal rigid sphere (Duda & Martens, 1998; Hartley & Fry, 1921; Shinn-Cunningham, Santarelli, & Kopco, 2000; Stewart, 1911). HRTFs also have been measured in the acoustic near field using a Knowles Electronic Manikin for Acoustic Research (KEMAR) (Brungart & Rabinowitz, 1999; Calamia & Hixson, 1997; Kopčo & Shinn-Cunningham, 2003). Distance judgments for lateral sounds were more accurate than for sounds in the median plane, consistent with HRTF measurements indicating that ILD varied with distance (Brungart et al., 1999). Low-frequency ILD cues are relatively robust to room reverberation, and perceived distance judgments for close sound sources may be more veridical in a reverberant room where DRR cues also are available in addition to ILD cues (Shinn-Cunningham et al., 2005). Results from a study by Kopčo and Shinn-Cunningham (2011) in a simulated reverberant room suggested that distance judgments could be explained on the basis of listeners using a fixed frequency-dependent mapping of DRR to distance, despite the presence of potential ILD cues. The authors suggested that further experiments were needed to establish whether ILD cues contribute to auditory distance judgments in reverberant space as well as in anechoic environments.

HRTF parallax also may be used to determine the distance to sound sources that are relatively close to the listener. Acoustic parallax occurs when a sound is relatively close to the head, introducing a difference between the angle of the source relative to the left ear, and the angle of the source relative to the right ear. Assuming that the direction from each ear can be determined, presumably using pinna cues that can be quantified by measuring HRTFs, the parallax angle can be calculated using the difference between the directions from each ear to the sound source. This varies as a function of source distance. For frontal sources, the parallax angle is larger for closer sources than for farther sources. Kim, Suzuki, Takane, and Sone (2001) obtained distance judgments using acoustic parallax with pink noise presented at virtual distances between 0.1 and 2 m; the stimuli were synthesized to remove level and DRR cues. Distance judgments increased with increasing source distance up to approximately 1 m, consistent with observations that HRTFs are almost independent of sound source distance beyond 1 m (Otani, Hirahara, & Ise, 2009). HRTF parallax may account for instances where participants were able to report auditory distance for frontally presented sounds at near distances even when level and ILD cues were unavailable (Ashmead et al., 1990).

### Dynamic cues

Acoustic flow information arising from the motion of sound sources and/or the listener can provide auditory distance information in two forms: that of acoustic tau and absolute motion parallax. Acoustic tau refers to the rate of change in sound level as the listener moves (Ashmead, Davis, & Northington, 1995). Estimates of acoustic tau can be related to distance estimates, as they are proportional when the velocity is constant (Zahorik et al., 2005). Although rate of change in level has been proposed to be the main cue used to specify acoustic tau, rate of change for other cues such as spectral content and binaural cues could potentially also provide useful information (Ashmead et al., 1995). Acoustic tau can also be expressed as the time to contact for a sound source that approaches the listener with a constant velocity; it is the distance divided by the velocity. Auditory time-to-contact estimates tend to be underestimated (Schiff & Oldak, 1990), consistent with underestimation of static auditory distance (Zahorik, 2002a; Zahorik et al., 2005). The second form of dynamic acoustic information, absolute motion parallax, is the change in angular direction of the sound source caused by movement of the source relative to the listener. An experiment in which static or moving observers judged distances to static 20-Hz pulse trains at distances between 2 and 6 m in a quiet outdoor environment showed that dynamic cues led to small improvements in distance accuracy (Speigle & Loomis, 1993). Ashmead et al. (1995) showed greater benefits of acoustic tau when the sound source was a noise burst of random intensity presented between 5 and 19 m from the participant, as accuracy at walking to the location of the sound increased when participants listened while walking compared with when they stood still. These studies suggest that dynamic cues benefit auditory distance perception for distances greater than 2 m. However, a study by Teramoto, Sakamoto, Furune, Gyoba, and Suzuki (2012) showed that, for acoustic near space, movement is detrimental to distance judgments. For tone bursts presented up to 1.5 m away, self-motion resulted in greater errors in judging distance than no self-motion.

Further investigations are necessary to investigate auditory distance perception abilities when both the sound source and the listener are moving. The use of dynamic auditory distance cues in this case will be limited, as various combinations of distance and path of motion can give rise to identical absolute motion parallax and acoustic tau, and use of acoustic tau is dependent upon the participant knowing the velocity of the translating source (Speigle & Loomis, 1993). It is currently unclear how useful dynamic cues are for relative distance judgements, as studies to date have utilized absolute distance judgement tasks only (Ashmead et al., 1995; Speigle & Loomis, 1993; Teramoto et al., 2012). It also is unclear how beneficial dynamic cues are in reverberant environments, as studies so far have used outdoor environments (Ashmead et al., 1995; Speigle & Loomis, 1993) or a corridor where sound-absorbing materials were placed on the walls (Teramoto et al., 2012).

For dynamic sound sources, a consistent asymmetry in distance judgments has been reported (Hall & Moore, 2003). For sounds that increase in level, simulating a looming source, listeners perceive a greater change in loudness than for sounds that decrease in level, simulating a receding sound source (Neuhoff, 1998); distance was not judged. This adaptive bias may be due to the perceived biological importance of looming sounds, which potentially indicate an oncoming threat or collision or successful acquisition of desirable objects or goals (Cappe et al., 2012). The bias has been observed for harmonic tones but not broadband noise (Ghazanfar, Neuhoff, & Logothetis, 2002; Neuhoff, 1998), possibly because tonal sounds are more likely to originate from a biological source (Ghazanfar et al., 2002). These findings are important, because they may indicate that approaching objects are treated with priority by the perceptual system and that the neural system has evolved to address this (Ghazanfar et al., 2002). However, Teghtsoonian, Teghtsoonian, and Canévet (2005) showed that perceptual judgments of the change in level of dynamic sounds may be influenced more by the end level of the stimulus than by the change in level and suggested that caution was needed in speculations regarding the evolutionary basis of biases for looming sounds.

Caution is needed in interpreting the results of some looming studies in terms of perceived distance, due to the use of relatively impoverished stimuli that may have given a limited impression of movement. In studies where the findings were interpreted in terms of perceived distance, the stimuli actually consisted of a non-moving sound that rose or fell in level by, e.g., 15 dB, and participant responses were given on an arbitrary “no change” to “large change” scale (Neuhoff, 1998; Seifritz et al., 2002). In addition, the use of headphones in some distance-perception studies may have resulted in the stimuli sounding as if they were located within the head. Binaural information helps to “externalize” sounds heard over headphones and reducing binaural information reduces perceived externalization (Catic et al., 2013; Hartmann & Wittenberg, 1996). Externalization (perceiving the sound as located outside versus inside the head) is related to but distinct from auditory distance perception (perceiving the sound to be located at a specific distance from the head). Externalization is a prerequisite for auditory distance perception. A sound that is externalized is usually perceived as being at a specific distance, but the precision with which the distance can be judged may vary depending on the available cues. Seifritz et al. (2002) presented sounds diotically (the same sound to each ear) over headphones and asked participants to report whether they perceived sound motion for rising, falling, and constant level tones and to rate the strength of apparent motion on a visual analog scale. Motion was perceived for most trials using rising or falling tones. However, they pointed out that the motion percept was not as compelling as might have been achieved by convolving the headphone signals with HRTFs or using an array of loudspeakers to generate a moving sound source in the free field. We are not aware of any studies that have quantified the perceptual bias for looming versus receding stimuli in terms of perceived changes in distance or that have investigated how distance cues other than level affect the perceptual bias for looming sounds.

The conditions in which the various auditory distance cues described so far can be utilized are summarized in Table 1.

**Table 1:** Summary of the conditions in which each auditory distance cue can be used. For each condition, a checkmark (√) indicates that the cue is available and can be used, a cross (x) indicates that the cue is not useful, and a question mark (?) indicates that the answer is currently unknown or unclear (see main text for further details). Frontal and lateral sources refer to sound position relative to the listener

Condition	Auditory distance cue				
	Level	DRR	Spectral cues	Binaural cues	Dynamic cues
Anechoic environment	√	x	√	√	√
Reverberant environment	√	√	√	?	?
Absolute distance judgements	x	√	x	√	√
Relative distance judgements	√	√	√	√	?
Frontal sources	√	√	√	?	√
Lateral sources	√	√	√	√	√
Peripersonal space	√	√	√	√	x
Extrapersonal space	√	√	√(>15 m)	x	√

### Stimulus familiarity

Experience with sound sources previously heard across a range of distances can increase the accuracy of perceived distance judgments, because listeners can compare spectral content and sound level at the ears with an internal estimate of the probable spectra and output power of the sound source. For example, a siren from a fire engine with a low level at the receiver’s ears is normally perceived to be far away, because sirens generally have a high output power (Philbeck & Mershon, 2002). Stimulus familiarity is particularly useful for stimuli that are consistent in overall level and spectral content, such as gunshots (Zahorik, et al., 2005). Internal estimates are more difficult to derive for dynamic or unpredictable stimuli. Two studies showed that repeated exposure to unfamiliar sounds increases the accuracy of auditory distance estimates. Coleman (1962) reported that participants’ accuracy improved over successive trials when judging distance to broadband noise bursts presented in a free field at distances between 2.7 and 8.2 m, and Mershon et al. (1989) found that estimates of distance for broadband noise bursts became more veridical over a series of five trials in a reverberant room at distances between 0.75 and 6 m. Shinn-Cunningham (2000) presented listeners with broadband noises up to 1-m away and observed that distance judgements became more veridical over the course of 3-5 days.

Familiarity with speech signals can benefit listeners when making distance judgments. The acoustic characteristics of speech change systematically as the vocal effort and output level of the speaker change, such as when the speaker is whispering, talking conversationally, or shouting. Whispered speech can be identified by its lack of voicing (Pickett, 1956), and shouted speech has relatively more high-frequency energy than conversational speech (Cheyne et al., 2009; Eriksson & Traunmüller, 2002). These changes allow listeners to estimate the distance to the talker by comparing the perceived production level of the speech to the intensity of the signal at the ear (Brungart & Scott, 2001). Several studies have shown that estimates of the distance of speech stimuli are generally quite accurate (Cochran et al., 1968; Gardner, 1969; von Békésy, 1949) and are more accurate than for unfamiliar stimuli, such as noise (Zahorik, 2002a). Estimates of the distance to a talker increase systematically at a fixed location as the speakers’ vocal effort increases from conversational to shouted, whereas distance estimates for whispered voices decrease (Brungart & Scott, 2001; Gardner, 1969). Philbeck and Mershon (2002) confirmed that the source familiarity effects observed in these studies were due to long-term knowledge of the typical characteristics of speech rather than experimental context (comparison of stimuli across trials), by showing that shouted voices were reported as farthest and whispered voices closest even upon first stimulus presentation. Thus, source familiarity can provide absolute auditory distance information (Mershon & Bowers, 1979).

Time-reversed speech is spectrally and temporally similar to normal speech but does not contain semantic information. McGregor, Horn, and Todd (1985) and Brungart and Scott (2001) found that the use of time-reversed speech reduced the ability to use vocal effort information in distance estimation. They suggested that listeners required phonetic information to interpret vocal-effort-based cues. Wisniewski et al. (2012) assessed distance discrimination by English speaking participants for stimuli that were lexically and phonetically familiar (English speech), phonetically familiar only (Bengali Speech), or both lexically and phonetically unfamiliar (time-reversed English and Bengali Speech). They found that while participants judged the distance of forward speech more accurately than that of backwards speech, accuracy did not differ between English and Bengali speech, suggesting that speech distance discrimination depends on phonetic rather than lexical familiarity.

### Effect of visual information on perceived auditory distance

Visual information can affect the perceived spatial location of sounds. The most famous example of this is the ventriloquist effect, where the speaker’s voice appears to come from the visual location of the source (Warren, Welch, & McCarthy, 1981). There have been reports that similar effects of visual capture occur for auditory distance judgments, described as the proximity effect (Gardner, 1968; Mershon et al., 1980; Zahorik, 2001). Hládek et al. (2013) presented audiovisual stimuli consisting of broadband noise bursts that were spatially congruent or incongruent with light-emitting diodes (LEDs) placed at distances between 44.5 and 349 cm from the participant in a dark reverberant room. When the visual stimuli were presented 30% closer to or farther away than simultaneous auditory stimuli, a ventriloquism effect occurred; participants reported a shift in the perceived location of the auditory targets towards the visual stimuli. A weaker ventriloquism aftereffect was also shown, where the shift occurred for audio-only trials that were interleaved with audiovisual trials. For localization in azimuth, the ventriloquist effect has been explained in terms of localization blur; the blur is usually much less for the visual than for the auditory modality. Alais and Burr (2004) reported that when visual localization was good, visual low-contrast Gaussian blobs captured the spatial location of sound clicks. When the visual stimuli were heavily blurred and visual localization was poor, the opposite effect was observed, and sound captured vision. This can be modelled as an optimal combination of visual and auditory spatial information, where each cue is weighted by an inverse estimate of its variability to produce a stimulus estimate with the lowest possible variance.

Visual estimates of distance are known to be better than auditory estimates (Da Silva, 1985; Loomis et al., 1998), and it is possible that visual capture based on localization blur explains the proximity effect. However, this requires experimental confirmation. Also, it is not yet clear what features are needed for the auditory and visual stimuli to be assumed by the perceptual system to be coming from the same source, when visual and auditory distance cues conflict. Audiovisual perceptual binding requires temporal synchrony, and participants may use visual and auditory distance cues to maintain perceptual synchrony despite the relatively low speed of sound relative to light (Alais & Carlile, 2005; Sugita & Suzuki, 2003). However, other work suggests that this is not the case (Heron et al., 2007).

### Combining auditory distance cues

An internal representation of distance to a sound source is built up by combining information from the various cues that are available. For example, it is not possible to make accurate distance judgments using a fixed mapping of DRR to distance, because mean DRR values corresponding to specific distances depend on source spectral content, on the characteristics of the room, and on whether the DRR is measured at the near- or far-ear relative to the sound (Kopčo & Shinn-Cunningham, 2011). Different cues vary in terms of reliability and are dependent on sound-source properties and the environment. Thus, the relative weighting of each cue in determining the percept of distance needs to be flexible. Zahorik (2002a) showed that the perceptual system did indeed weight level and DRR cues flexibly to produce a single distance percept depending on the stimulus and the angular position of the sound source relative to the listener. However, the processes underlying cue combination and weighting associated with other cues, such as spectrum and binaural information have yet to be explored. Kopčo and Shinn-Cunningham (2011) suggested that the auditory system may optimally combine DRR and ILD information in reverberant rooms to improve the precision of distance estimates to lateral sound sources. However, this requires further testing.

### Nonperceptual factors

An emerging body of evidence shows that the auditory system is adaptively biased in order to overcome or avoid immediate threats. As described, events of potential biological importance, such as looming (approaching) stimuli (Neuhoff, 1998, 2004; Seifritz, et al., 2002) may affect distance perception. Also, nonperceptual factors, such as fear, may play a role (Gagnon, Geuss, & Stefanucci, 2013). However, the parameters that determine the extent to which perceived distance is affected, such as the degree of adaptive bias produced by various auditory distance cues, have yet to be explored in detail.

According to the superordinate view of emotions proposed by Tooby and Cosmides (2008), emotions influence other processes, such as perception to allow efficient implementation of an evolved function, such as avoiding immediate threats. If an emotion, such as fear, alters perception by making a sound appear closer, it may promote action to cope with the possible threat. To investigate this, Gagnon et al. (2013) tested whether participants in a fearful state perceived sounds to be closer than participants in a neutral state. In one condition, blindfolded participants were asked to reach to a commercial dog training clicker, heard at distances that were either within reach or out of reach. Participants in a fearful state judged the distance to the target to be closer than participants in a neutral state, judging targets to be reachable at distances that were 33% farther than for the neutral group. These findings are consistent with a study of Siegel and Stefanucci (2011) showing that tones are perceived to be louder by participants in a fearful state than by participants in a neutral state and are supported by similar findings in the visual domain (Stefanucci et al., 2012; Sugovic & Witt, 2013).

The emotional valence of the sound source itself also might affect auditory distance judgments. The perceived urgency of sounds is related to sound source spectrum, with increased urgency associated with higher-frequency content (Hellier et al., 2002), and higher-frequency content is associated with closer perceived distance (Butler et al., 1980; Coleman, 1968; Little et al., 1992; von Békésy, 1938). Sounds perceived to be urgent, such as a cry for help, thus might be judged to be closer than neutral sounds.

## Development of auditory distance processing

Infants’ perception of auditory distance has generally been assessed by measuring how their actions match spatial information conveyed by proximal sensory stimulation, such as reaching to grasp sound-producing objects (Ashmead, Clifton, & Perris, 1987; Clifton, Perris, & Bullinger, 1991; Litovsky & Clifton, 1992) or moving to avoid approaching objects (Freiberg, Tually, & Crassini, 2001). Such actions suggest that the sound-producing object is perceived in spatial terms relative to the location of the infant (van der Meer, Ramstad, & Van der Weel, 2008). The literature on developmental aspects of auditory distance perception has not been reviewed previously and is discussed in this section.

Clifton et al. (1991) showed that infants were able to distinguish between objects in near and far space on the basis of sound by 6 months of age. Sounds were presented in the dark either within reach at 15 cm, or out of reach at 60 cm. Infants reached more frequently towards the location of the sound when positioned within reach than when out of reach. This was replicated in a follow-up study by Litovsky and Clifton (1992), who further demonstrated that infants correctly discriminated between near and far sounds regardless of whether or not sound level was roved to prevent it from providing a useful cue. This suggests that infants use other cues than sound level when judging distance, in contrast to adults tested in the same study, who relied primarily on level cues and whose performance worsened when level was roved.

Using a conditioned head turn technique, Morrongiello, Hewitt, and Gotowiec (1991) showed that by 6 months of age infants were better at discriminating approaching than receding stimuli. They also demonstrated that responses on trials where changes in distance occurred were greater than responses on trials using non-moving sounds that increased or decreased in sound level, suggesting that distance cues other than changes in level were utilized. Freiberg et al. (2001) used a more direct auditory looming paradigm to assess relative distance perception using sound level cues. They hypothesized that if infants used changing sound level to perceive changes in distance to the sound source, then they would engage in more defensive avoidance behavior for auditory stimuli that increased rather than decreased in level. Consistent with this hypothesis, avoidance behavior, as measured by amount of backward body pressure exerted by the infant, was associated with level increases but not decreases.

Two studies have investigated whether infants are able to coordinate auditory and visual distance information (Morrongiello & Fenwick, 1991; Walker-Andrews & Lennon, 1985). Walker-Andrews and Lennon (1985) showed 5-month-old infants two videos side by side of automobiles approaching or receding. The videos were paired with a soundtrack of a lawn mower either increasing or decreasing in level. Infants looked preferentially at the video that matched the soundtrack for approaching stimuli only. A second study, also using a preferential-looking procedure, showed that 9-month-old infants were able to coordinate visual and auditory depth information for both approaching and receding stimuli (Morrongiello & Fenwick, 1991). Visual information of a drum-beating toy was presented on two screens with auditory information that matched one of the screens. The toy was shown moving horizontally in depth or stationary. Five-month-old infants only reliably looked preferentially at the stationary toy paired with the stationary sound stimulus, suggesting that they did not recognize that changes in sound level indicated that the distance of an object was changing. Nine-month-old infants preferentially looked at the screen that matched the auditory stimulus for which the depth changed. The authors suggested that the extended time period between perceiving auditory distance at approximately 6 months (Morrongiello et al., 1991) and coordinating it with visual depth was due to younger infants having difficulty recognizing that increases and decreases in sound level accompany an object moving in depth, possibly because sounds can vary in level independent of source distance. The discrepancy between their findings and those of Walker-Andrews and Lennon (1985) was attributed to the increased salience of visual depth cues in the prior study, recruiting the attention of 5-month-old infants and aiding coordination of audiovisual depth. Overall, these studies suggest that by 9 months of age, infants are able to coordinate visual depth information with auditory distance cues and hence could use visual information in order to calibrate auditory space.

Infants younger than 11 months are able to discriminate increments in sound level of 3 dB and decrements of 6 dB (Bull, Eilers, & Oller, 1984; Sinnott & Aslin, 1985; Tarquinio, Zelazo, & Weiss, 1990). This suggests that changes in level are potentially usable as a distance cue. By 3 years of age, children increase their vocal intensity as the distance from a listener increases (Johnson et al., 1981), indicating that they have at least limited knowledge of the intensity losses due to sound propagation. Further work is needed to determine more closely how auditory distance perception develops, using conditions where individual distance cues are controlled and tested independently of other cues. One possible avenue of further research is to investigate how auditory distance is calibrated for normally sighted, early- and late-onset blind individuals, by investigating the accuracy of absolute auditory distance judgments longitudinally from infancy to adulthood, to establish how internal representations of auditory space are generated and maintained when visual calibration cues are unavailable. The internal representation of auditory distance and its calibration are discussed in more detail in the following section.

## Internal representation of distance to a sound source: calibration of auditory peripersonal and extrapersonal space and perceptually guided locomotion

For normally sighted listeners, the calibration of auditory distance is thought to be achieved primarily using visual signals, as localizing a sound generally involves directing the gaze toward the sound source to identify it and to obtain further information about it (Lewald, 2002a). Indeed, Perrott, Saberi, Brown, and Strybel (1990) hypothesized that “The primary function of the auditory spatial system may be to provide information that allows the individual to redirect the eyes in order to bring the fovea into line with an acoustically active object.” As a result, auditory space is constantly updated using visual and motor feedback when developing an internal spatial representation of the surroundings to align auditory and visual spatial representations (Lewald, 2013). For listeners with severe visual losses, an alternative method of calibrating auditory space involves using sensory-motor feedback, such as touching the sound source. A computational sensorimotor model has been developed demonstrating that auditory space can be learned using motor actions without the need for visual cues (Aytekin, Moss, & Simon, 2008). These approaches have emphasized sound localization in azimuth, and calibration of auditory distance judgments has not received as much scientific study. Questions remain regarding how well internal representations of auditory distance can effectively guide the locomotor system in the absence of visual cues, and how the calibration of auditory distance differs in peripersonal space and beyond it.

Visual information can be used to calibrate auditory distance as it provides more accurate spatial information than audition (Da Silva, 1985; Loomis et al., 1998). Auditory distance judgments are more accurate when visual range information regarding the whole scene is available, even if the sound source itself is visually occluded (Calcagno et al., 2012; Zahorik, 2001). Anderson and Zahorik (2014) presented participants with virtual sound sources, simulated using binaural room impulse responses (BRIRs) measured using distances of 0.3 to 9.8 m between a loudspeaker and a KEMAR in a concert hall. They showed that auditory distance judgments were more accurate and less variable when matched to a congruent visual stimulus consisting of a photograph of the measurement loudspeaker taken from the position of the head of the KEMAR.

For accurate distance judgments, the auditory system has to scale appropriately the internal representation of the available distance cues so that the perceptual distance matches the external distance as closely as possible. For example, a reduction of 6 dB in sound level in an anechoic environment should correspond to a doubling of the internal representation of source distance. As described earlier, the distances of remote sounds are systematically underestimated (Fontana & Rocchesso, 2008; Kearney et al., 2012; Zahorik, 2002a; Zahorik et al., 2005), suggesting that internal auditory distance representations in extrapersonal space are not well calibrated (visual cues may be insufficient to accurately calibrate auditory distance in far space) or that the auditory cues do not permit accurate judgments of distance in far space.

In peripersonal space, auditory distance can be calibrated using vision and by touching the sound source. However, auditory distance judgments for sounds presented within this region show overestimation of sound source distance (Zahorik, 2002a; Zahorik & Wightman, 2001), suggesting that auditory distance representations are not well calibrated in peripersonal space either. Internal representations of auditory distance are integrated with tactile information in the space immediately surrounding the head (Farnè & Làdavas, 2002; Graziano, Reiss, & Gross, 1999) or hand (Canzoneri, Magosso, & Serino, 2012; Serino et al., 2007; Serino et al., 2011). In a study by Serino et al. (2007), participants were given an electrical stimulus on their right index finger, paired with a burst of white noise either close to the hand or at a distance of 125 cm. Normally sighted participants showed faster reaction times to tactile stimulation when the concurrent sounds were close than when they were far, indicating the existence of a peri-hand space in which auditory distance cues are integrated with tactile information. Integration of distance information within peripersonal space helps to protect the body from injury (Graziano & Cooke, 2006), as it is within this area that the individual can reach or act on objects, or avoid approaching threats (Canzoneri et al., 2012). Further work is needed to investigate which auditory distance cues are integrated with tactile information in peripersonal space and how they are weighted in this process.

Accurately calibrated distance perception is needed to complete everyday navigation tasks, such as locomotion through the environment (Rand et al., 2011). This requires space to be represented accurately in relation to action capabilities. Vision provides relatively accurate internal spatial representations of an object relative to the body, allowing the CNS to guide locomotor patterns in a feedforward manner (see Higuchi, Imanaka, and Patla (2006) for a review). For audition, it has been shown that auditory distance cues can provide internal spatial representations with sufficient fidelity to allow the CNS to guide locomotion to a target sound source in the absence of vision (Loomis et al., 1998; Russell & Schneider, 2006). Motor responses require the production of a motor program that is adapted to the auditory signal (Wanet & Veraart, 1985). Thus, it might be hypothesized that distance judgments made when standing still would be more accurate than motor responses to auditory distance (i.e., judgements that involve movement, such as pointing or walking to the perceived position of the sound source). However, verbal judgments of distance are generally not more accurate than locomotive responses. Loomis et al. (1998) compared verbal estimates of sound-source distance to perceptually directed walking for speech stimuli presented between 4 and 16 m from the participant. The results showed underestimation of the distance to far sounds, consistent with absolute distance judgments reported in other studies (Zahorik et al., 2005). The verbal and motor estimates were generally consistent, although greater variability was observed for the verbal estimates. No difference was found between gymnasts and non-gymnasts, suggesting that internal representations of auditory distance are associated with systematic error even for groups for whom action is highly skilled. Russell and Schneider (2006) showed that verbal distance judgments were less accurate than when participants walked to the perceived location of a loudspeaker producing 0.7-kHz tones for distances between 1.6 and 3.1 m, under conditions where the sound source varied in both distance and azimuth and reverberation cues were available. Overall, these results suggest that internal representations of auditory distance can be used by the CNS to guide locomotion. Further work is needed to establish whether internal auditory representations of distance are sufficient to allow the CNS to plan and guide locomotion in a feedforward manner.

## Effect of visual loss on auditory distance perception

For totally blind individuals or those with light perception only, audition provides the primary source of information regarding distance to a target in extrapersonal space. Auditory distance information is also of paramount importance to those with partial visual losses, such as age-related macular degeneration (AMD), retinitis pigmentosa (RP), or glaucoma, which can severely reduce central or peripheral visual spatial information.

**Fig. 2:**
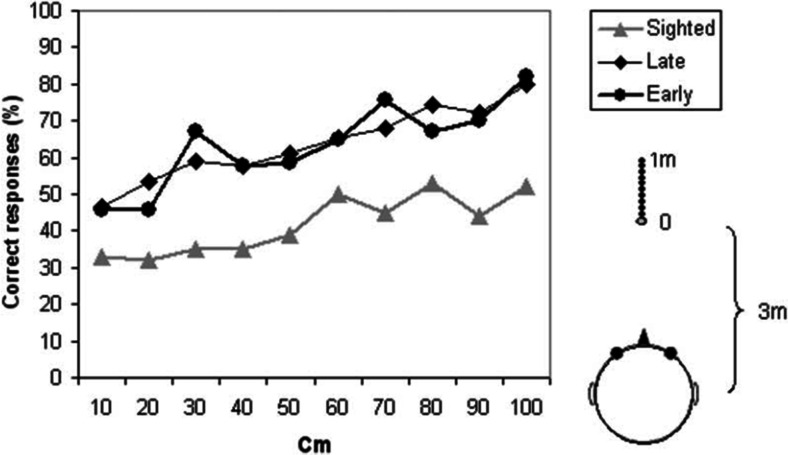
Percentage of correct responses in an auditory distance discrimination task, for various distances between a reference sound positioned 3 m from the participant and a comparison sound source positioned farther than the reference. Responses are shown for three groups: normally sighted (grey triangles), early-onset blind (black circles), and late-onset blind (black diamonds). From “Early- and Late-Onset Blind Individuals Show Supra-Normal Auditory Abilities in Far-Space,” by P. Voss, M. Lassonde, F. Gougoux, M. Fortin, J. Guillemot, and F. Lepore, 2004, *Current Biology*, *14*, p. 1736. Copyright 2004 by Elsevier Ltd. Reprinted with permission

Voss et al. (2004) showed that both early- and late-onset blind participants could discriminate the distance of broadband white noise bursts presented 3-4 m away in a reverberant room, whereas normally sighted participants could not (Fig. 2). Using virtualization techniques to simulate anechoic and reverberant rooms, Kolarik, Cirstea, and Pardhan (2013b) showed that blind participants used level and DRR cues more effectively than normally sighted or partially sighted participants to discriminate the distance of broadband white noises presented between 1 and 8 m from the participant. These studies suggest that significant auditory compensation occurs following full visual loss and that it provides measurable benefits across a range of acoustic environments for relative auditory distance perception.

In contrast, absolute distance accuracy is not better for blind than for sighted participants, either in peripersonal or extrapersonal space. A study requiring absolute distance judgments of 800-Hz tones presented between 18 and 62 cm from the participant showed poorer performance for early-blind participants than for late-blind and sighted controls (Wanet & Veraart, 1985). In another study, blind participants with age of onset up to 8 years showed greater errors in motor distance judgments (pointing at the perceived location of the sound) for white-noise stimuli presented within reaching distance than for sighted participants (Macé, Dramas, & Jouffrais, 2012). Lai and Chen (2006) asked blind and sighted participants to report perceived distance to a musical tone or a telephone ring, presented at a fixed distance of 3 m in front of them. On average, sighted participants showed lower errors than blind participants, but the difference was not significant. Kolarik et al. (2013c) presented participants with speech sounds at virtual distances between 1.2 and 13.8 m in an anechoic room. Normally sighted participants underestimated the distance to far sounds, consistent with previous work (Zahorik, et al., 2005). Early-blind participants underestimated the distance to far sounds and also overestimated the distance to close sounds. These studies suggest that severe visual loss results in lower accuracy for absolute auditory distance judgments.

Why do blind participants show enhanced performance for relative distance but deficits for absolute distance? One possible explanation is that blindness results in distorted internal spatial representations of distance, which adversely affect absolute judgments of distance. For sound-producing objects in peripersonal space, the tactile modality could in principle be used to calibrate auditory space. However, the findings of Wanet and Veraart (1985) suggest that this is not sufficient to accurately calibrate audition for those with early-onset blindness. Furthermore, blind individuals are rarely if ever able to calibrate distance in extrapersonal space using audiomotor feedback by walking to a sound source and touching it (Kolarik et al., 2013d). Without accurately calibrated auditory distance, internal spatial representations of distance may become distorted, adversely affecting blind individuals’ abilities to judge absolute distance. In contrast, relative distance judgments rely on comparisons between internal representations of distance, and are not adversely affected by any warping or distortion of the mapping between the internal representation and distance, provided the mapping remains monotonic. In this case, better discrimination of internal representations of distance can lead to superior relative distance judgments.

In an experiment of Schiff and Oldak (1990), normally sighted participants either viewed a film paired with a soundtrack of looming objects that disappeared before reaching them or heard the sound track only. They were required to press a button to predict when the object would have reached them. Early-blind participants performed the task with the sound track only. For the sound track only condition, the early-blind group was more accurate than the normally sighted group, although a tendency for underestimation of distance was still observed. The authors suggested that the superior performance of the early-blind group was due to greater perceptual learning and attention to acoustical approach in the early-blind group.

To our knowledge, auditory distance perception abilities using spectral cues have not yet been tested for blind participants. However, there have been studies of the ability to localize in azimuth and elevation. In a study by Voss et al. (2011), participants made “same” or “different” judgments of pairs of noise bursts filtered with HRTFs, where the first noise had a simulated azimuth between ±60° and 0°, and the second noise had a simulated azimuth either at the same position or ±30° away from the first stimulus. Early-blind participants were better able to perform the task than normally sighted participants. Lessard et al. (1998) assessed the localization of broadband noise bursts, including a condition where one ear was occluded, necessitating the use of spectral cues for localization. The sounds were presented from loudspeakers with azimuths up to ±78°. For monaural listening, localization performance for half of the early-blind participants fell outside the normal range and was superior to that of sighted participants. Similar findings were reported by Doucet et al. (2005), who reproduced the findings of Lessard et al. (1998), and further showed that the performance of blind participants was affected by the elimination of spectral cues by covering the pinna with acoustic paste more than for sighted participants.

Other studies have reported that blind participants are worse than sighted participants when performing elevation localization judgments that require spectral cues (Lewald, 2002b; Zwiers, Van Opstal, & Cruysberg, 2001). In a study by Lewald (2002b), bursts of broadband noise were presented from loudspeakers positioned in the median plane along a frontal arc of ±31°. On average, pointing responses were less accurate for blind participants than for normally sighted participants. The ability of blind listeners to use spectral cues may depend on whether those cues are appropriately calibrated for the different tasks. Localization in azimuth may be calibrated using sensorimotor information, but this may be more difficult for localization in elevation since sound sources varying in elevation may be beyond reaching range.

Blind participants have been shown to demonstrate enhanced abilities to discriminate fine spectral changes for perceiving pitch (Gougoux et al., 2004; Wan et al., 2010) and spatial position based on spectral profile (Voss et al., 2011), and in principle this might allow better use of spectral cues for auditory distance judgments. Enhanced sensitivity to spectral information could benefit blind listeners when making distance judgments in peripersonal space, where spectral cues provide distance information (Brungart, 1999; Kopčo & Shinn-Cunningham, 2011). It also is possible that the increased DRR sensitivity for distance discrimination in extrapersonal space reported for blind listeners by Kolarik et al. (2013a) was due to enhanced abilities to process spectral cues, because these have been shown by Larsen et al. (2008) to be the main factor mediating DRR discrimination.

Auditory distance estimates are related to the perceived dimensions of the room in which the sound is heard, because in the absence of visual cues the perceived position of the farthest sound source indicates the minimum possible distance to the far wall. One study found a significant correlation between room size judgments and maximum perceived sound source distance (Kolarik et al., 2013d) for speech, music, and noise stimuli presented at virtual distances between 1.2 and 13.8 m. The relationship was stronger for blind than for sighted participants, suggesting that blind participants rely mainly on the perceived distance of the farthest sound when estimating room size, whereas sighted participants rely at least partly on alternative sources of information. Because the study was correlational and therefore does not imply causation, it is possible that perceived room size affects auditory distance judgments. For example, if the listener is informed that sound sources are being heard in a large room, in the absence of vision the range of their judgments of distance might be extended to far sources. However, this has yet to be confirmed.

Serino et al. (2007) showed that blindness results in extended multisensory peripersonal space in blind cane users. They showed that for sighted participants, reaction times to tactile stimuli were increased when concurrent sounds were presented located close to the hand compared with when sounds were presented farther away, demonstrating that a limited region of multisensory peripersonal space is present close to the hand where audition and touch are integrated. However, reaction times to tactile stimuli paired with far sounds were faster for blind cane users than for normally sighted participants when a cane was held. Because blind people use a cane to integrate auditory distance information with tactile cues in far space, these results indicate that blindness and associated long-term use of a cane generate a new representation of multisensory peripersonal space near the end of the cane, similar to that for the area near the hand for normally sighted people. This new representation may help blind people to avoid collisions (Serino et al., 2007) and is consistent with the proposal that the coding of peripersonal space serves to protect the individual from harm (Graziano & Cooke, 2006).

In addition to the auditory distance cues described above, some blind people use echolocation from self-generated sounds and ambient sound cues to judge the distance to silent objects. Echolocation involves producing bursts of sound and listening to the returning echoes, and it is used by dolphins and bats to detect and localize nearby objects. Both blind and sighted people can be trained to use echolocation, and currently known cues include the energy of the echoes relative to the emitted sound, the time delay between the emitted sound and the echoes, spectral changes, binaural high-frequency differences, and differences in the reverberation pattern within a reverberant room. On average, blind people tend to be better echolocators than sighted people. For reviews, see Kolarik, Cirstea, Pardhan, and Moore (2014) and Stoffregen and Pittenger (1995). Echolocation has been found to be effective for distances up to approximately 2-4 m, although precision decreases with increasing distance (Rowan et al., 2013; Schenkman & Nilsson, 2011; Wallmeier & Wiegrebe, 2014). A recent study showed that distance discrimination thresholds based on echolocation were below 1 m for blindfolded sighted participants and for an expert blind echolocator, for reference distances between 0.75 and 4 m in a virtual environment (Wallmeier & Wiegrebe, 2014). Thresholds were smallest (approximately 20 cm) for the closest reference distance of 0.75 m and increased with increasing reference distance. These thresholds were lower than those for distance discrimination between pairs of external sound sources in a virtual environment as measured by Kolarik et al. (2013b), which were approximately 0.8 and 1.5 m for reference distances of 2 and 5 m, respectively, for blind participants, and larger for sighted participants. These results suggest that echolocation can provide reasonably accurate distance information and may contribute to the calibration of auditory space (Wallmeier & Wiegrebe, 2014). Ashmead et al. (1998) showed that ambient sound could be used to provide distance information about silent objects by blind children, who utilized the buildup of low-frequency ambient sound near large objects or walls for guidance during locomotion. Sound pressure buildup could be used to detect a wall from a distance of approximately 1 m, enabling the children to walk parallel to the wall, and avoiding the need to tap a cane periodically to check the wall position.

Sensory substitution devices (SSDs) are electronic travel aids, some of which are based on echolocation, that have been developed to help blind individuals perceive the distance to silent objects. Visual-to-auditory SSDs use an ultrasound or optical source and a receiver to detect signal reflections from obstacles. The range is set by the user or the device itself and information is converted to an auditory signal that can effectively convey distance from the object to the user (Hughes, 2001; Sohl-Dickstein et al., 2014) and inform locomotion (Kolarik et al. 2014a, b). Other devices that translate visual patterns into sound have been demonstrated to provide the user with effective distance information and guide motor performance, including the “prosthesis substituting vision with audition” (PSVA, Renier et al., 2005), the vOICe (the middle three letters stand for “oh I see"; Meijer, 1992), and the EyeCane (Maidenbaum et al., 2013; Maidenbaum et al., 2014). SSDs have high potential to increase the spatial awareness of the blind and have been utilized in the laboratory to identify multimodal brain areas for depth processing (Renier et al., 2005) and to investigate neural plasticity arising as a consequence of visual loss (De Volder et al., 1999). However, SSD sounds may interfere with the perception of environmental sounds that provide distance cues as well as other spatial information, and this may be a factor contributing to the relatively low use of SSDs by blind individuals (Roentgen et al., 2009).

## Effects of hearing loss and hearing aid processing on auditory distance perception

In contrast to investigations of the effects of hearing loss on localization in azimuth (see Keating & King, 2013 for a review), there are relatively few psychophysical studies and no neuronal studies of how auditory distance perception is affected by hearing loss. Effects of hearing aid processing, which potentially may distort available auditory distance cues, also have received relatively little attention. Adverse effects of hearing impairment or hearing-aid processing on auditory distance perception may be compensated to some extent by visual depth information for normally sighted listeners. However, considerable difficulties may occur in situations where vision is degraded, and although hearing loss is an important consideration for blind listeners, this area of enquiry is currently under-researched. The effect of sensory loss and hearing aid processing on auditory distance perception is especially important for older people, because visual and auditory losses are more prevalent in this group.

Akeroyd et al. (2007) compared the effectiveness of level and DRR distance cues combined with DRR alone for normal-hearing and hearing-impaired participants. They measured distance discrimination for sentence pairs at virtual distances between 1 and 8 m. Hearing-impaired participants generally performed as well as normally hearing participants when both cues were available, although hearing-impaired participants performed more poorly for simulated distances greater than 5 m. Hearing-impaired participants performed more poorly when the level cue was made unavailable by fixing the overall level of the sounds, suggesting deficits in the ability to discriminate distance using DRR. The scores obtained with DRR cues alone were correlated with self-reported auditory distance perception abilities.

Most modern hearing aids include amplitude compression that applies high gain for low-level sounds and low gain for high-level sounds. This increases the audibility of low-level sounds without making intense sounds uncomfortably loud (Moore, 2008). However, alterations to sound level due to hearing aid processing may alter the cues utilized to perceive distance accurately (Simon & Levitt, 2007). Amplitude compression alters level cues and can affect DRR cues by reducing gain for high-level direct sound while providing high gain for low-level reverberant sound. However, for continuous speech, the reverberant tail only occurs in isolation, during pauses in speech. Thus, adverse effects of hearing aid processing might be expected to be small or negligible. This was found in a study by Akeroyd (2010), who investigated distance discrimination for continuous speech using the design of Akeroyd et al. (2007) described above, with level and DRR cues both available. Akeroyd (2010) did not find any adverse effects of hearing-aid compression produced by the participants’ own hearing aids. As the participants were experienced hearing aid users, it is possible that they acclimatized to the effects of their own hearing aids on sound level. It also is possible that no adverse effects were found, because the amount of amplitude compression was small or because the gain changed too slowly to affect the DRR. Effects of hearing-aid compression might be observed for absolute distance judgments rather than the relative distance task utilized in the study (Akeroyd, 2010).

Hearing aid compression may affect the use of ILD distance cues (Simon & Levitt, 2007) even for continuous speech, and although this has not yet been directly assessed, there is evidence that compression distorts ILD cues for localization in azimuth. Musa-Shufani et al. (2006) tested normally hearing and hearing-impaired participants with narrow-band one-third octave wide noise signals centered at 500 and 4000 Hz in a localization task. Hearing aids were simulated with linear processing or fixed amounts of fast or slow compression. Fast compression was found to increase JNDs in ILD compared with linear processing.

In summary, hearing loss adversely affects the use of DRR cues, although the use of level cues remains relatively unaffected (Akeroyd et al., 2007). Hearing aid compression does not affect distance discrimination when level and DRR cues are available (Akeroyd, 2010). Further work is needed to expand upon these findings, in particular to investigate how hearing loss affects absolute distance judgments, the use of spectral and binaural distance cues, and the effects of bilateral versus unilateral fitting. Although the effect of hearing loss on the use of distance cues other than level and DRR has yet to be evaluated, it is likely that the use of high-frequency spectral content for estimating distance to far sounds will be affected, because hearing loss often is greater at higher than lower frequencies (Moore, 2007). Use of binaural cues for near-distance judgments may be impaired due to reduced frequency selectivity caused by the broadening of auditory filters with hearing loss, which reduces the ability to obtain ITD and ILD information from within narrow frequency bands (Moore, 2007). Due to the importance of accurate spatial awareness for blind individuals, if detrimental effects on distance perception as a consequence of hearing-aid amplitude compression were to be identified, it is possible that blind individuals would derive greater benefits from hearing aids with linear processing. However, this has yet to be investigated.

## Neuronal bases of auditory distance perception

It often is assumed that auditory processing occurs along functionally separate pathways within the auditory cortex (AC), organized along similar lines to the “what” and “where” pathways in the visual cortex, such that spatial information is processed in a posterior stream of the AC (Ahveninen et al., 2013; Rauschecker & Scott, 2009; Rauschecker & Tian, 2000; Recanzone & Cohen, 2010). Horizontal sound direction changes have been shown to activate posterior nonprimary AC regions, including the planum temporale (PT) and superior temporal gyrus (STG) (Ahveninen et al., 2006). Auditory distance may be processed in areas, including the PT and STG (Kopčo et al., 2012), within a dedicated network that includes the ventral premotor cortex (Graziano et al., 1999) and anterior cingulate cortex (ACC) (Wisniewski et al., 2012). There is currently little neural data regarding how visual loss affects neural processing of auditory distance. However, work involving learning of the distance to sounds suggest the recruitment of occipital areas (Chan et al., 2012; Tao et al., 2013). These results parallel findings for localization in azimuth, which have shown that visually deafferented areas are functionally recruited for spatial auditory processing in the event of visual loss (for reviews, see Collignon et al., 2009; Voss & Zatorre, 2012).

Mathiak et al. (2003) utilized magnetoencephalography (MEG) to investigate neural correlates of auditory distance processing. A series of white noise bursts with deviants in level and (as a control) duration were presented in various conditions, simulating sound sources at distances that included 0 m (i.e., within the head) and 2 m. The results suggested that the right AC plays a dominant role in the processing of level and DRR distance cues. Deviants in level evoked a larger response over the right supratemporal plane than the left, but this activation decreased when reverberation was present. In macaque monkeys, multimodal neurons in ventral premotor cortex have been found to represent distances in peripersonal space (Graziano et al., 1999), and it is possible that the purpose of spatial representation in this area is to guide head and arm movements in relation to targets that are within reaching and grasping distance (Graziano & Gross, 1998; Moore & King, 1999). Graziano et al. (1999) reported that many of the neurons they tested changed their response when the level was changed, but 59% of the neurons tested coded distance independent of level. The authors suggested that cues other than level are used to process distance in the near field, including reverberation and spectrum, and these might determine the responses of the neurons. Further experiments are needed to analyze the relative influence of these potential cues.

**Fig. 3:**
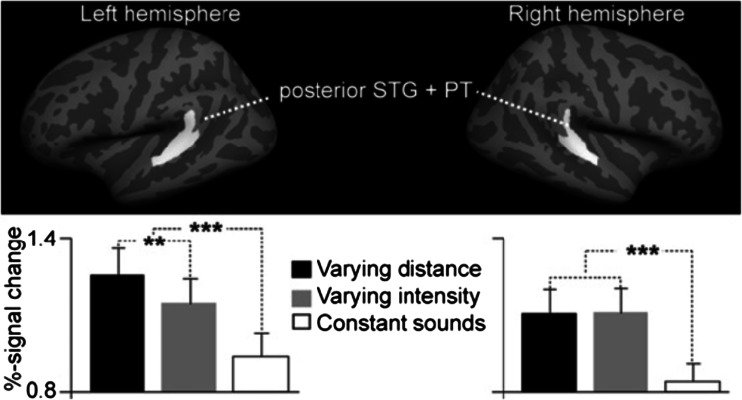
Posterior nonprimary auditory cortex activations for hypothesis-based region-of-interest (ROI) analyses during auditory distance processing. Increases in left posterior auditory cortex ROI activity occurred when the distance of the sound source was varied versus when the sound level (intensity) was varied. This suggests the presence of neurons with distance representations that are independent of level in the posterior nonprimary auditory cortices. Increased activity was observed in both hemispheres during varying intensity versus constant sounds. Error bars represent +1 standard error of the mean. From “Neuronal representations of distance in human auditory cortex,” by N. Kopčo, S. Huang, J. Belliveau, T. Raij, C. Tengshe, and J. Ahveninen, 2012, *Proceedings of the National Academy of Sciences of the United States of America*, *109*, p. 11019. Copyright 2012 by National Academy of Sciences. Reprinted with permission

In another study focusing on peripersonal space (Kopčo et al., 2012), sensitivity to acoustic distance changes independent of level was observed in neural populations in the PT and posterior STG (Fig. 3). Wisniewski et al. (2012) utilized electroencephalography (EEG) to investigate the neural mechanisms underlying distance perception for familiar or unfamiliar speech sounds under conditions where level cues were minimized. They reported that cortical regions responded in different ways depending on sound familiarity across a network that included the ACC, suggesting that working memory and attentional processing were implicated when familiarity was a factor in distance judgments.

As described earlier, using monaurally presented noise at virtual distances between 10 and 160 cm, Kim et al. (2015) showed that inferior colliculus neurons in the rabbit either increased or decreased firing rates monotonically as AM depth increased, but only when the virtual environment was reverberant and AM was present. These findings suggest that neural sensitivity to AM depth, combined with the distance-dependent reduction of AM depth in reverberant environments, may provide a mechanism for the neural coding of monaural distance. Altmann et al. (2013) demonstrated that loudness constancy occurred in conditions where reverberation was comparatively strong, but not in weak reverberation conditions. In contrast, the accuracy of auditory distance judgments was similar in strong and weak reverberation conditions, suggesting dissociation between loudness constancy and distance perception. MEG recordings suggested that the right middle temporal and inferior anterior temporal cortex play a role in the representation of loudness, reflecting perceptual loudness constancy, while superior temporal areas are implicated in sound distance perception processing (Kopčo et al., 2012).

It has been speculated that the greater biological salience of approaching than retreating sounds may have a neural basis (Ghazanfar et al., 2002). A neural network involving the superior temporal sulcus, middle temporal gyrus, right premotor cortex, and right temporoparietal junction has been implicated in distance processing for dynamic sound sources (Seifritz et al., 2002), and it is likely that the right PT plays a role in auditory distance processing for dynamic sounds (Hall & Moore, 2003).

Several studies that used sound-to-distance learning paradigms involving SSDs have provided insight regarding the neural networks involved in auditory distance perception for the blind. Renier et al. (2005) trained normally sighted blindfolded participants to use a visual to auditory SSD for perceiving distance to objects in peripersonal space. Using positron emission topography (PET), they showed that occipitoparietal and occipitotemporal areas were activated while using the device, suggesting that areas of the visual cortex are somewhat multimodal and can be recruited for perceiving distance by audition. Chan et al. (2012) tested early-blind and normally sighted controls using an echo-based SSD to judge distance for objects placed 1-5 m from the SSD. Functional magnetic resonance imaging (fMRI) showed that learning was mediated by a parieto-frontal network that involved the hippocampus and the cuneus in the striate cortex. The neural network for normally sighted individuals involved reduced activity in the occipital lobe and hippocampus, and increased activity in the frontal and temporal lobes. Tao et al. (2013) measured fMRI responses from early- and late-onset blind groups when using auditory spatial information from an SSD signal to locate objects at 1.5-, 2.5-, or 3.5-m distance at various azimuths. They also evaluated participants’ visuospatial working memory abilities. Both groups showed activation in middle occipital gyrus, superior frontal gyrus, precuneus, and precentral gyrus when localizing sounds. However, the groups differed in activation of the right middle occipital gyrus and left superior frontal gyrus. In the early-blind group, sound localization performance was correlated with BOLD responses in the right middle occipital gyrus only. In the late-onset group, BOLD responses in the left superior frontal gyrus were correlated with sound localization performance and visuospatial working memory abilities. The results suggest that early-onset visual loss results in cross-modal plasticity that recruits occipital areas for processing auditory spatial information, including distance, whereas spatial processing occurs in prefrontal areas involving visuospatial working memory for those with late-onset visual loss.

## Concluding remarks and suggestions for further research

Despite the advances in our understanding of auditory distance perception, this area remains relatively under-researched compared with sound localization in azimuth. There are currently gaps in our understanding of the effects of partial visual loss, dual loss, occluding objects, background noise, and multiple sources on perceived distance to sounds, and accuracy of distance judgements for sounds located behind or vertically relative to the listener. These areas require further study and are discussed below.

Very little is currently known regarding the effects of partial non-correctable visual loss on auditory distance perception, and perceptual processing in this population remains under-researched relative to that for those with total visual losses (Occelli, Spence, & Zampini, 2013). Kolarik et al.(2013b) found no difference in auditory distance discrimination using level, DRR, or both cues between a partially sighted group and a normally sighted group, whereas enhanced performance was found for those with full visual loss. The possibility that partial visual loss may affect auditory distance perception is suggested by work indicating that partial sensory loss can affect abilities in unimpaired modalities (Bavelier, Dye, & Hauser, 2006; Després, Candas, & Dufour, 2005; Dufour & Gérard, 2000; Hoover, Harris, & Steeves, 2012). However, reports of the effects of partial visual loss on localization in azimuth have been conflicting. Enhanced auditory localization in azimuth was reported for participants who had lost one eye (Hoover et al., 2012) or were myopic (Després et al., 2005; Dufour & Gérard, 2000). However, Lessard et al. (1998) found no evidence of sensory compensation among a partially sighted group for localization in azimuth.

The consequences of auditory impairment for spatial awareness are of high importance to those with severe visual loss, due to their increased reliance on auditory cues (Simon & Levitt, 2007). However, we are not aware of any studies that have assessed auditory distance perception for those with dual losses. DRR sensitivity for distance discrimination is enhanced following severe visual loss (Kolarik et al., 2013b) but is reduced following hearing impairment (Akeroyd et al., 2007). Research is needed to establish whether compensation associated with blindness provides a “buffer” for diminished hearing abilities associated with ageing or whether hearing loss degrades auditory distance discrimination for both normally sighted and blind individuals.

The presence of occluding objects between the listener and a sound source causes changes in the sound received by the listener. This may affect the perceived distance of the sound source. However, investigation of the effect of occluding objects on perceived auditory distance is currently lacking. A recent study showed that the presence of a sonic crystal composed of an array of rigid plastic cylinders in air, placed between the listener and a sound source (one-third-octave noise bands, with the center frequency ranging from 0.5 to 2 kHz) resulted in an illusion of proximity, where the sound was perceived to be closer to the listener (Spiousas et al., 2015). This was likely due to changes in acoustic distance cues caused by the presence of the crystal, because sound level, DRR, and interaural cross-correlation values were substantially increased. One study investigated perceived distance to passing trains either outside or inside a dwelling, where the walls attenuated the overall inside sound by approximately 13 dB and attenuated the high frequencies even more due to building insulation (De Coensel et al., 2012). Participants based their distance judgments on their perception of the expected sound level outdoors and not on the attenuated sounds actually heard indoors, suggesting that participants were able to take into account the knowledge that the sound source was occluded. It often is the case that the listener does not have such prior knowledge, such as when listening to a talker originating outside the visual field who is obstructed by another person. Further investigation is needed to establish how occluding objects influence distance perception.

Mershon et al. (1989) reported that perceived auditory distance for sound sources presented at distances between 0.75 and 6 m in rooms with high or low reverberation decreased as the background noise level increased. With the exception of this study, the effects of noisy environments on auditory distance perception are unknown, as is the impact of multiple sound sources. It seems feasible that auditory distance information could help to segregate sound sources in acoustically complex conditions, such as when background noise or reverberation is present. This could help focus attention and improve identification of the sound source, including in “cocktail party” situations (Bronkhorst, 2015; Cherry, 1953; Haykin & Chen, 2005; Kidd et al. 2005).

Investigation of the effect of background noise and multiple sound sources could have considerable relevance to judgments of the distance of vehicles by blind individuals, who have to rely on sound to detect gaps in traffic when trying to cross a road. Although the studies described above indicate that blind individuals may develop enhanced relative distance perception in quiet environments (Kolarik et al., 2013b; Voss et al., 2004) and for dynamic sounds (Schiff & Oldak, 1990), benefits may be limited in noisy multisource situations. Guth et al. (2013) showed that, compared with normally sighted participants, blind participants road-crossing judgments at a single-lane roundabout were more risky, particularly when traffic volume was high. Judgments were slower, and fewer opportunities for crossing were taken. Safer judgments were made when the crossing location was farther from the roundabout, where overall noise levels were reduced. In high-volume traffic, blind participants reported difficulties when judging the approach of vehicles against a background of other traffic noise, suggesting that noise impaired distance judgments. Additional effects of multiple sound sources were observed immediately after a vehicle passed the participant, as blind participants had to wait several seconds for the sound of the vehicle to decay to judge the approach distance of the next vehicle. Further investigations of the effect of multiple sound sources and background noise on the usability of relevant auditory distance cues in similar situations, such as overall level and spectral information, potentially could increase safety for blind participants when crossing the path of traffic.

It is currently unknown how accurate distance perception is for sounds located behind the listener, where accurate spatial hearing would be beneficial from an evolutionary perspective, as vision does not provide information in this situation. Evolved navigation theory (ENT) proposes that “distance perception is a primary mechanism for relaying fitness costs over evolutionary time into differential navigation decisions (Jackson, 2009).” The theory predicts that observers overestimate visual distances over navigationally costly surfaces and may be applicable to vertical as well as horizontal auditory distance judgments. The accuracy of judgments of vertical auditory distance, such as when judging the height or depth of sound sources, also is currently unknown, and possible links between fear state and auditory distance judgments, or effects of acrophobia (fear of heights) on distance judgments, have not been explored. Such research might provide insights regarding how superordinate mechanisms affect auditory distance perception, and the origin of individual differences in auditory distance tasks.

In summary, there is now a considerable and growing body of evidence indicating that auditory distance perception provides important spatial information that guides behavior across a wide range of different acoustic environments. Recent work has provided valuable insights regarding the neural processes underlying auditory distance perception, the consequences of sensory loss, and how perceived auditory distance is adaptively biased in order to overcome or avoid immediate threats. However, gaps remain in our understanding of auditory distance processing, and issues, including the effects of hearing loss, partial visual loss, dual loss, occluding objects, background noise, and multiple sources on perceived auditory distance, remain to be explored.
